# Subclinical Hypothyroidism among Chronic Kidney Disease Patients Admitted to Nephrology Department of a Tertiary Care Centre: A Descriptive Cross-sectional Study

**DOI:** 10.31729/jnma.8128

**Published:** 2023-04-30

**Authors:** Rupesh Kumar Shreewastav, Asok Kumar Ghosh, Rahul Yadav, Anup Katwal, Shailendra Shrestha

**Affiliations:** 1 Department of Biochemistry, Nobel Medical College Teaching Hospital, Biratnagar, Morang, Nepal; 2 Department of Nephrology, Nobel Medical College Teaching Hospital, Biratnagar, Morang, Nepal

**Keywords:** *chronic kidney disease*, *thyroid stimulating hormone*, *thyroxine*, *triiodothyronine*

## Abstract

**Introduction:**

Chronic kidney disease is a condition, which worsens the quality of life in many ways including thyroid disorder in many cases. The aim of the study was to find out the prevalence of subclinical hypothyroidism among chronic kidney disease patients admitted to the Nephrology Department of a tertiary care centre.

**Methods:**

A descriptive cross-sectional study was carried out on the patients diagnosed with chronic kidney disease at a tertiary care hospital from 15 May 2022 to 10 October 2022 after getting ethical approval from the Institutional Review Committee (Reference number: 621/2022). Pre-designed proforma was used to collect demographic data like age, sex, height and weight. Blood samples of the patients were analysed for thyroid function tests (triiodothyronine, thyroxine and thyroid stimulating hormone levels) by chemiluminescence immunoassay. Convenience sampling was used. Point estimate and 95% Confidence Interval were calculated.

**Results:**

Out of 156 study participants with chronic kidney disease, subclinical hypothyroidism was present in 34 (21.79%) (15.31-28.27, 95% Confidence Interval) patients.

**Conclusions:**

The prevalence of subclinical hypothyroidism amongst chronic kidney disease patients was found to be lower than in other similar studies conducted in similar settings.

## INTRODUCTION

Chronic kidney disease (CKD) is a condition that is quickly spreading throughout the world's population.^1^ According to estimates, the disease affects over 9% of the world's population, especially in developing nations.^2,3^

Maintaining healthy renal function regulates thyroid hormone metabolism and elimination.^4,5^ The kidney is a crucial end-organ for thyroid hormonal activity.^6^ CKD has a variety of effects on thyroid function. Reports suggest that CKD is largely associated with thyroid dysfunction; especially most common is primary and subclinical hypothyroidism.^7,8^

The main objective of the study is to find out the prevalence of subclinical hypothyroidism among chronic kidney disease admitted to the Nephrology Department of a tertiary care centre.

## METHODS

A descriptive cross-sectional study was conducted at Nobel Medical College Teaching Hospital (NMCTH), Biratnagar, Morang, Nepal on the patients diagnosed with CKD from 15 May 2022 to 10 October 2022. The study was conducted after receiving ethical approval from the Institutional Review Committee (IRC) of the NMCTH (Reference number: 621/2022). All CKD patients who were admitted to the nephrology ward during the study period were included in this study. Patients with other comorbidities and who do not give consent were excluded. A convenience sampling method was used. The sample size was calculated by using the formula:


$$\begin{array}{ll} \text{n} & ={\text{Z}}^{2}\times \frac{\text{p}\times \text{q}}{{\text{e}}^{\text{2}}}\\ & =1.96^{2}\times \frac{0.272\times 0.728}{0.07^{2}}\\ & =156 \end{array}$$

Where,

n = minimum required sample size
Z = 1.96 at 95% Confidence interval (CI)
p = prevalence of subclinical hypothyroidism taken from the previous study as, 27.2%^9^
q = 1-p
e = margin of error, 7%

The calculated final sample size was 156. Predesigned proforma was used to gather personal information like age, sex, height, and weight. The formula used to determine body mass index (BMI) is BMI= Weight in Kg/ (Height in m).^2^ The level of triiodothyronine (T3), thyroxine (T4) and thyroid stimulating hormone (TSH) was estimated in the blood samples of these patients by chemiluminescence immunoassay (CLIA) in a fully automatic analyzer (Maglumi 800) at the clinical laboratory services, NMCTH.

The level of T3/T4 in the blood sample was estimated by a competitive chemiluminescence immunoassay. The sample, ABEI labelled anti-T3/T4 monoclonal antibody, buffer and solution of the magnetic microbeads coated withT3/T4 antigens were incubated at 37° C. T3/T4 present in the sample competed with T3/T4 antigen immobilised on the magnetic microbeads for a limited number of binding sites on the ABEI labelled anti-T3/T4 antibody forming immune-complexes. After washing, the starter was added to initiate a chemiluminescent reaction. The light was measured by a photomultiplier within 3 seconds as relative light units, which was inversely proportional to the concentration of T3/T4 present in the sample.

The TSH level in the blood sample was measured by a sandwich chemiluminescence immunoassay. The sample, ABEI labelled anti-TSH monoclonal antibody, magnetic microbeads coated with another anti-TSH monoclonal antibody were mixed and incubated at 37°C, forming sandwich immune complexes. After washing, the starter was added to initiate a chemiluminescent reaction. The light was measured by a photomultiplier within 3 seconds as relative light units, which was proportional to the concentration of TSH present in the sample.

The collected data were entered into and analysed using Microsoft Excel version 2010. Point estimate and 95% CI were calculated.

## RESULTS

Out of 156 patients with chronic kidney disease, subclinical hypothyroidism was present in 34 (21.79%) (15.31-28.27, 95% CI) patients. Among them, 14 (41.17%) were male and 20 (58.82%) were female. The mean age was 53.47±16.33 years. The mean value of body mass index (BMI) of the CKD patients with subclinical hypothyroidism was 25.31±5.28 kg/m^2^ (Range: 19.50-40.90) (Table 1).

**Table 1:** Baseline characteristics of study participants with chronic kidney disease having subclinical hypothyroidism (n= 34).

**Characteristics**	
Gender	n (%)
Male	14 (41.17)
Female	20 (58.82)
**Age group (years)**	
<40	8 (23.52)
41-60	14 (41.17)
61-80	10 (29.41)
>80	2 (5.88)
	**Mean±SD**
**Biochemical parameters**	
Serum urea (mg/dl)	140.31±61.46
Serum creatinine (mg/dl)	8.35±5.04
eGFR (ml/min)	10.53±7.57

The mean value of T3 among patient with subclinical hypothyroidism was 3.01±0.35 (Table 2).

**Table 2:** Value of thyroid hormones among the CKD patients with subclinical hypothyroidism (n = 34).

Hormones	Mean±SD
T3 (pg/ml)	3.01±0.35
T4 (ng/dl)	1.06±0.24
TSH (uIU/ml)	12.76±22.54

**Figure 1:**
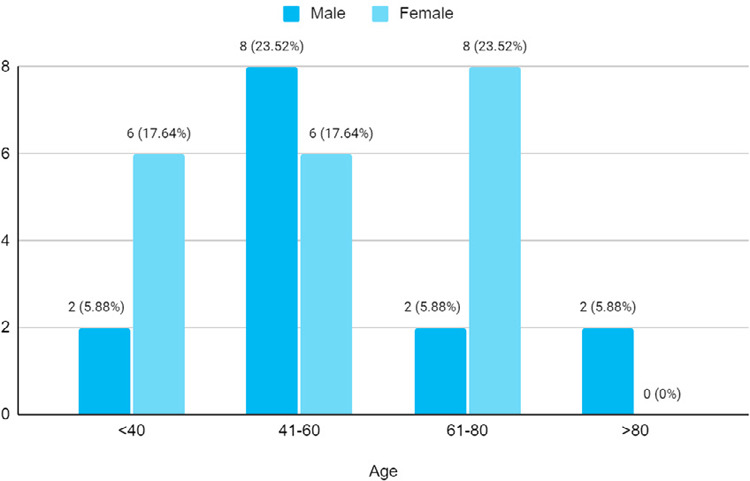
Sex-wise distribution of CKD patients with subclinical hypothyroidism in various age groups (n= 34).

The maximum number 8 (23.52%) of male patients were reported from age group 41-60 years; whereas the maximum number 8 (23.52%) of female patients was reported from age group 61-80 years (Figure 1).

## DISCUSSION

Out of 156 CKD patients, subclinical hypothyroidism was observed in 21.79% of patients. Amongst them, the number of males and females in the study was 14 (41.17%) and 20 (58.82%) respectively with a mean age of 53.5 years. Females were more sufferers than males. A higher prevalence of subclinical hypothyroidism among CKD patients was observed in a study carried out in BPKIHS, Dharan, Nepal, which showed that subclinical hypothyroidism was seen in 27.2% of CKD patients. The mean age of all patients was 44.1±16.4 years with 53.8% male and 46.1% female.^9^ In another research conducted on hemodialysis patients in western Nepal revealed that 26.6% of them had both subclinical and clinical hypothyroidism.^10^ Another study from North India reported that subclinical hypothyroidism was observed in 39.9% of total chronic kidney disease patients.^11^ A research from Oman revealed a result, reporting a prevalence of subclinical hypothyroidism of 62.9% among CKD patients.^12^ In literature, it is reported that when compared to people with euthyroidism, those with hypothyroidism had a CKD or (95% CI) of 1.25 (1.21-1.29) and therefore concluded that people with CKD were more likely to have hypothyroidism.^13^ Almost similar finding was observed in large cohort research, 22% of CKD patients with eGFRs ≤60 had hypothyroidism.^7^

A lower rate of prevalence of subclinical hypothyroidism among CKD patients was observed in a study carried out in Saudi Arabia, which reported that amongst CKD patients, 16.9% suffered from subclinical hypothyroidism.^14^ A study from Italy concluded that in people with CKD who do not need chronic dialysis, subclinical primary hypothyroidism is a rather frequent disease (18%) and is independently linked to steadily declining estimated GFR in a sizable population of unselected outpatient adults.^15^ A study was conducted in the Japanese population with CKD, which showed the prevalence of subclinical hypothyroidism as 14.9% among the patients.^16^

In the present study, we also observed the mean value of T3, T4 and TSH amongst the patients of CKD with subclinical hypothyroidism. The mean±SD value of T3, T4 and TSH in the blood of the CKD patients with subclinical hypothyroidism was 3.01±0.35 pg/ml, 1.06±0.24 ng/dl and 12.76±22.54 uIU/ml respectively. In a study, it has been reported that the mean±SD value of TSH among CKD patients with subclinical hyperthyroidism was 9.01±4.40 uIU/ml respectively. Similarly, the mean±SD value of T4 was 1.22±0.13 ng/ dl.^11^ In the same way, in another report, it was seen that the mean value of TSH in CKD patients with subclinical hyperthyroidism was 5.40 uIU/ml.^14^ A study from Bangalore, India reported the mean±SD value of TSH as 7.23± 4.21 among CKD patients.^17^ The mean±SD value of TSH was reported as 7.15± 5.94 among CKD stage-IV patients in Gujarat, India in research.^18^

Thyroid dysfunction was observed in CKD patients ages more than 40 in the current study. In the age group 4160 and 61-80, the patients diagnosed with subclinical hyperthyroidism were 41.17% and 29.41%. In a study, it was observed that CKD patients from 36-45 years and 45-55 years were 48% and 32% respectively.^18^

The data were only gathered from one centre and at one specific moment, which is one of the study's limitations. If data on CKD patients had also been collected from other centres in Nepal and the patients had been followed up for a longer period, the findings would have been more generalisable.

## CONCLUSIONS

The prevalence of subclinical hyperthyroidism among CKD patients was lower than the other similar studies done in similar settings. Subclinical hypothyroidism was observed more in females and also in advanced-age patients with chronic kidney disease.
